# Getting to the Heart of the Matter: The Role of Regulatory T-Cells (Tregs) in Cardiovascular Disease (CVD) and Atherosclerosis

**DOI:** 10.3389/fimmu.2019.02795

**Published:** 2019-11-28

**Authors:** Caraugh J. Albany, Silvia C. Trevelin, Giulio Giganti, Giovanna Lombardi, Cristiano Scottà

**Affiliations:** ^1^British Heart Foundation Centre, School of Cardiovascular Medicine and Sciences, King's College London, London, United Kingdom; ^2^Peter Gorer Department of Immunobiology, School of Immunology and Microbiological Sciences, King's College London, London, United Kingdom; ^3^Department of Internal Medicine, University of Milan, Milan, Italy

**Keywords:** regulatory T cells (Tregs), cardiovascular disease (CVD), hyperlipidemia, hypertension, atherosclerosis

## Abstract

Cardiovascular diseases (CVD) are the leading cause of mortality worldwide. Atherosclerosis is directly associated with CVD and is characterized by slow progressing inflammation which results in the deposition and accumulation of lipids beneath the endothelial layer in conductance and resistance arteries. Both chronic inflammation and disease progression have been associated with several risk factors, including but not limited to smoking, obesity, diabetes, genetic predisposition, hyperlipidemia, and hypertension. Currently, despite increasing incidence and significant expense on the healthcare system in both western and developing countries, there is no curative therapy for atherosclerosis. Instead patients rely on surgical intervention to avoid or revert vessel occlusion, and pharmacological management of the aforementioned risk factors. However, neither of these approaches completely resolve the underlying inflammatory environment which perpetuates the disease, nor do they result in plaque regression. As such, immunomodulation could provide a novel therapeutic option for atherosclerosis; shifting the balance from proatherogenic to athero-protective. Indeed, regulatory T-cells (Tregs), which constitute 5-10% of all CD4^+^ T lymphocytes in the peripheral blood, have been shown to be athero-protective and could function as new targets in both CVD and atherosclerosis. This review aims to give a comprehensive overview about the roles of Tregs in CVD, focusing on atherosclerosis.

## Introduction

According to the World Health Organization [https://www.who.int/news-room/fact-sheets/detail/cardiovascular-diseases-(cvds)], cardiovascular diseases (CVD) are a group of disorders affecting the heart and blood vessels; which include coronary heart disease (ischemic heart disease), angina, myocardial infarction, congenital heart diseases (e.g., tetralogy of Fallot, ductus arteriosus, transposition of great vessels, tricuspid atresia), hypertension, stroke (e.g., ischemic or hemorrhagic), heart valve diseases (e.g., regurgitation or stenosis), cardiomyopathy (e.g., heart failure with dilated or hypertrophic cardiomyopathy or with preserved ejection fraction), and vascular dementia.

Atherosclerosis is a common cause of CVD and is characterized by slow progressing inflammation in conductance and resistance arteries, in which there is an accumulation of cholesterol-containing low-density lipoprotein (LDL) particles beneath the endothelial layer (1). These lipid accumulations often take several decades to become symptomatic. Lesions associated with the disease can be found in the aorta as early as the first decade of life. Aging, genetic, and environmental factors lead to the spread of these lesions, they can be found in sites such as coronary arteries in the second decade of life, and cerebral vessels in the third and fourth decades of life (1).

Hypertension and hyperlipidemia are key risk factors for arteriosclerosis. As a result, statins (HMG CoA reductase inhibitors) have been widely used in CVD patients (2). Meta-analysis studies have suggested that for each 1 mmol/L reduction in LDL levels corresponds to a 22% decreased in the risk of stroke and coronary heart disease (3). However, data from surveys, registries, and insurance claims indicate that adverse effects of statins are common, which discourage patients from continuing therapy at recommended doses. Additionally, the underlying chronic inflammation of the blood vessel(s) is not completely resolved by statins.

Several studies have shown that the immune system is activated in atherosclerosis. Such observations indicate the possibility that selective suppression of proatherogenic or activation of athero-protective immune mechanisms may represent novel approaches for disease treatment. In recent years regulatory T-cells (Tregs) have emerged as crucial players in modulating both the innate and adaptive immune responses (4). Impaired Treg function and decreased frequency has been associated with the progression of atherosclerosis (5–7). Furthermore, adoptive transfer of such cells in animal models for atherosclerosis has been shown to be protective (7). Therefore, Tregs could be important targets in atherosclerosis and understanding their functions in this context is fundamental to driving future therapies.

## Pathogenesis of Atherosclerosis

**Figure 1:**
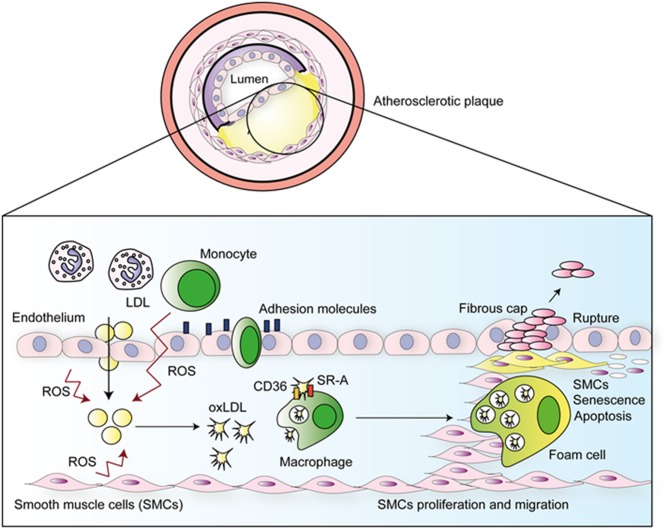
Atherosclerotic disease progression. ROS produced by ECs, SMCs, neutrophils, and macrophages oxidized LDL in the sub-endothelial space. Emigrated monocytes become macrophages which uptake oxLDL, subsequently forming foam cells, that are unable to migrate to arterial lumen, accumulate in the sub-endothelial space and die forming a large plaque with a necrotic core. Rupture of this plaque leads to thrombus formation.

The pathogenesis of atherosclerosis is illustrated in Figure 1. The disease is initiated by the passive diffusion of LDL into the arterial intima, this occurs preferentially in regions of higher blood turbulence or parts where the sites of endothelial damage (1, 8). Following diffusion into the sub-endothelial space, LDL is able to bind proteoglycans via apolipoprotein B-100 (ApoB100), and subsequently becomes permanently retained (9). The sequestered LDL undergoes oxidative modification forming oxLDL which causes aggregation and increased proteoglycan binding (8). Oxidation of LDL is mediated by reactive oxygen species (ROS) produced by smooth muscle cells (SMCs), endothelial cells (ECs), neutrophils, and macrophages (9, 10). These events are potentiated by production and release of monocyte chemotactic protein-1 (MCP-1) and macrophage colony stimulating factor (m-CSF), which, respectively, attracts circulating monocytes to the plaque and activates them to release more ROS, nitric oxide (NO), and pro-inflammatory cytokines, such as TNF-α and IL-1β (1, 11). In a positive feedback loop, ROS induces expression of TLRs in ECs, which perpetuates the inflammatory response via the expression of adhesion molecules, which cause circulating monocytes and other leukocytes to enter the tissue via trans-endothelial migration. Monocytes become differentiated into macrophages (8) which, once present within intima layer of the arteries, engulf oxLDL (8) through the scavenger receptors SR-A and CD36 (1). This uptake leads to the formation of foam cells, which have compromised migratory capacity. Consequently, these cells accumulate in the intima and die, resulting in the formation of a plaque with a necrotic core (1).

Growth factors and cytokines released by ECs and macrophages induce multiple effects including phenotypic changes within vascular SMCs, from the quiescent “contractile” phenotype state to the active “synthetic” state, that can migrate to and proliferate within the intima. The migratory and proliferative capacities of VSMC's increase the size of the atherosclerotic plaque. Some of the emigrated VSMCs become less differentiated, senescent, or undergo apoptosis, which contributes to plaque instability and rupture (12). This leads the formation of a traveling thrombus which can occlude smaller arteries, resulting in myocardial infarction (MI) or ischemic stroke (13).

## Atherosclerosis and T-Cells

Both the adaptive and innate arms of the immune system are involved in the development of atherosclerosis. As demonstrated in Figure 1, innate responses occur first in a non-antigen-specific manner (14). However, modified self-molecules such as oxidized LDL (oxLDL), β2 glycoprotein 1, Lipoprotein A (LP(a)), heat-shock proteins (HSP), and glycosylated proteins from the blood vessels extracellular matrix (collagen and fibrinogen) have been described as antigens, thus activate T-cell responses during atherosclerosis (9). Furthermore, foreign antigens including bacteria such as *Porphyromonas gingivalis* (15) and *Chamydia pneumoniae* (16), and viruses such as enterovirus (17) and cytomegalovirus have also been associated with atherosclerosis; potentially as causative or bystanders participants, adding yet another layer of intricacy to fully understanding the pathophysiology of atherosclerosis.

Antigen presentation by dendritic cells (DCs) during atherosclerosis is a complex matter. Early research, which aimed to elucidate the role of DCs in atherosclerosis relied on CD11c as the identifying cell surface marker. Functionally, high-phagocytic activity was demonstrated for CD11c^−^CD11b^+^MHCII^+^ macrophages that efficiently engulfed lipids, whereas CD11c^+^MHCII^+^ DCs present in the aorta were shown to display strong immune stimulatory capacities; being pivotal for T-cell activation and inflammation. However, in recent years it has become apparent that CD11c can also be expressed by MHCII^+^ monocytes and macrophages, and an ontogenetic view of cell lineages has defined DCs as a hematopoietic lineage distinct from other leukocytes (18). Thus historic studies addressing the role of CD11c^+^ (MHCII^+^) DCs may have also unintentionally included other cell types, such as monocytes/macrophages, therefore the term APC may have been more appropriate (19).

APCs travel to draining lymph nodes and present antigens for recognition by T-cells (14). Such antigen recognition results in clonal expansion of both CD8^+^ and CD4^+^ T-cells. CD4^+^ T-cells can secrete cytokines such as IL-17 (20) and IFN-γ (21), which facilitate the inflammatory process. Furthermore, IL-4 and IL-13 production by CD4^+^ T-cell activation leads to B-cell activation, clonal expansion, and subsequent immunoglobulin production (14). Antibodies appear to play a prominent role in atherosclerosis, arising due to increased number of immunogenic neo-epitopes which are typically present in the disease (14).

The atherosclerotic plaque consists of a heterogeneous population of cells, debris and extracellular matrix components (1). CD4^+^ T-cells can be divided in different subsets according to their capacity to support the type of immune response and cytokine production. Th1 cells are the most abundant T-cells and in the context of atherosclerosis, promote disease progression (1). These cells secrete IFN-γ which promotes lesion development and destabilization leading to the plaque rupture (1). Additionally, IFN-γ activates monocytes causing continuation of the response. Th2 and Th17 cells have also been found in atherosclerotic lesions but at lower frequencies. Th2 cells release IL-4, IL-5, IL-13, and support B-cell activation and antibody production (21). IL-4 induces the expression of the transcription factors GATA-3 via STAT-6 activation, and stimulates Th2 cell differentiation leading to upregulation of IL-5 which inhibits Th1 differentiation and therefore IFN-γ production (8, 21). As a result of this regulatory role Th2 cells were initially assumed to be beneficial in the setting of atherosclerosis. However, recent evidence indicates that these cells may be both helpful and disadvantageous depending on disease stage and/or lesion site. Th17 cells produce IL-17 which is a pro-inflammatory mediator. Th17 development is promoted by TGFβ in the presence of IL-6 and IL-23 (21). Similar to Th2, Th17 cells have been reported to have both positive and negative roles in atherosclerosis (21). Th17 cells produce factors such as IL-6, IFNγ, and granulocyte-macrophage colony-stimulating factor (GM-CSF), which are proatherogenic. However, opposing this proatherogenic role, Th17 cells can also convert into other cell types such as Tregs; by gaining expression of forkhead box P3 (FOXP3) and can subsequently exert suppressive effects on effector Th1 and Th2 cells.

## Regulatory T-cells (Tregs)

Tregs have been shown to be present within atherosclerotic plaques (22). Human Tregs were initially characterized as CD4^+^CD25^+^ T-cells in 2001 by several groups (23–26) based on the 1995 finding that murine Tregs constitutively express CD25 (27). These adaptive immune cells comprise 5–10% of all peripheral CD4^+^ T-cells (28), and play an indispensable role in the adaptive immune system being responsible for both immune homeostasis and maintaining self-tolerance (4).

## Treg Development

Tregs can be sub-divided into two main classes depending on their developmental origin: thymic Tregs (tTreg) and peripherally induced Tregs (pTreg). tTregs develop in the thymus, an environment where tTregs with a high affinity for self-antigens are positively selected for maturation (29). Once active, these cells can migrate out of the thymus and into the peripheral tissues and lymph nodes (28). Conversely, pTregs develop via antigenic stimulation from conventional CD4^+^ T-cells in the periphery.

## Subsets of pTregs

There are subsets of Tregs generated in the periphery which have specific phenotypes and a mechanism of action which is cytokine-dependent: Tr1 and Th3. Unlike tTregs, which arise as a separate sub-lineage from T-cell precursors in the thymus, Tr1 and Th3 derived from conventional peripheral Th0 cells and interact with and are susceptible to modulation by dendritic cells (30).

Tr1 cells are a population pTregs which are induced by sustained TCR engagement via chronic antigenic stimulation in the presence of high levels of IL-10. These cells act to induce and maintain peripheral tolerance. They do not exhibit constitutive FOXP3 expression, instead its expression is induced upon activation. Tr1 cells produce predominantly IL-10, whereas Th3 (also known as Tr2) predominantly produces TGF-β (30). Th3 cells differentiate in an antigen-non-specific manner and are identifiable via their expression of latency-associated peptide (LAP), IL-4 production and low CD25 expression, moderate levels of GITR and CTLA-4. Tr1 cells can produce IFN-γ and do not express detectable levels of GITR and low levels of CTLA-4 and CD25 (30).

Human Tregs consist of a heterogeneous population; characterized by the expression pattern of a vast range of cell surface molecules (27). Despite this variation, suppressive Tregs share the expression of certain “common” surface molecules such as CD4, CD25, and FOXP3 (31). FOXP3 is the transcription factor which is considered to be the lineage defining molecule, it's essential for both cell maturation and function (4).

## Suppressive Mechanisms

**Figure 2:**
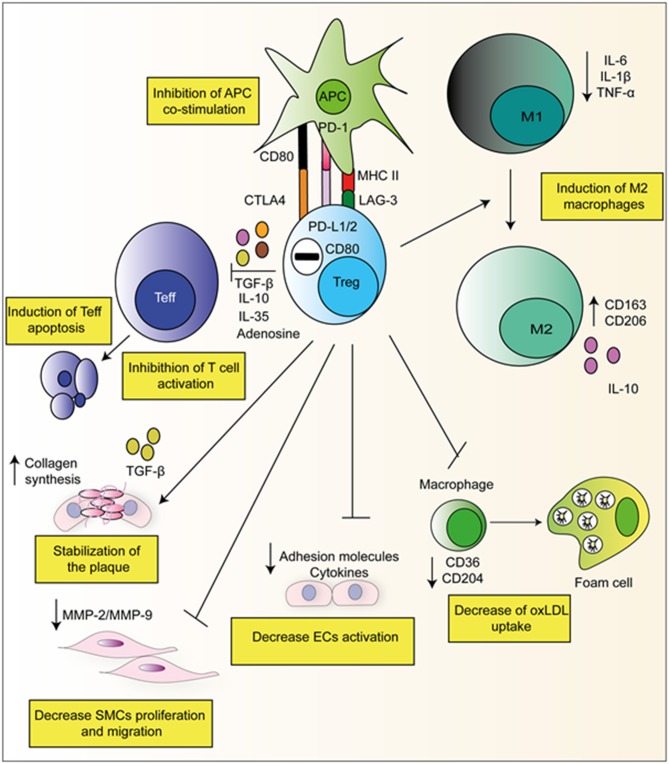
Suppressive mechanisms of Tregs in atherosclerosis. Tregs can directly produce suppressive cytokines and degradative enzymes such as perforin and granzyme that leads to apoptosis. Furthermore, Tregs have been observed to have direct effects on APC's via interaction with via CTLA-4, PD-L1/2, and LAG-3. They can also skew monocyte class switching, encouraging anti-inflammatory M2 macrophages formation which produced collagen and stabilizes the plaque. They can also decrease foam cell formation via the down-regulation of CD36 and CD204.

The key role of Tregs is to suppress both the adaptive and innate immune system. Tregs achieve this via the utilization of both direct and indirect pathways. In a direct manner, Tregs themselves elicit an immune response upon a target cell by for example, the secretion of suppressive cytokines such as IL-10, TGFβ, and IL-35 (23). In an indirect way by expressing higher levels of CD25, Tregs compete with T-effector cells (Teffs) for IL-2, which limits their proliferation. Tregs also express CD39/CD73 on their surface which produces adenosine from ATP, and activates adenosine receptors A2 on Teffs, which has inhibitory properties (23). Figure 2 gives examples of Treg suppressive mechanisms which are relevant in context of atherosclerosis.

## Evidence Implicating Tregs in Atherosclerosis

There has been a growing interest about the role about Tregs in atherosclerosis, with many studies aiming to better understand their significance. In 2006 using the ApoE^−/−^ mouse model, Ait-Oufella et al. demonstrated that CD4^+^CD25^+^ Treg deficiency is associated with a significant increase in atherosclerotic lesion size (5). This indicated for the first time that endogenous CD4^+^CD25^+^ Tregs play a protective role in atherogenesis (5). Further evidence came the following year, in 2007 Mor et al. (6) demonstrated that the adoptive transfer of wild type (WT) Tregs into ApoE^−/−^ mice resulted in a significant reduction in aortic sinus plaques as compared to the control mice (6). Furthermore, Tregs from ApoE^−/−^ mice we shown to have decreased *in vitro* suppressive ability (6). Then, studies in mice strongly suggest that defective Tregs may enable disease progression.

In 2013 Kligenberg et al. (7) demonstrated that deletion of FOXP3^+^ Tregs results in a 2.1-fold increase in plaque size in mice. FOXP3 was selectively depleted in the presence of diphtheria toxin using the DREG system in LDLR^−/−^ mice. This selective depletion resulted in increase in plasma cholesterol and VLDL levels and enhanced plasma enzyme activity of lipoprotein lipase, hepatic lipase, and phospholipid transfer protein (7). Importantly, in addition to induced changes within the cellular composition of the atheroma, Treg depletion also resulted in differences in genes controlling lipid metabolism in the liver and decreased the liver levels of sortilin, which may contribute to impairing intracellular cholesterol transport and increase the plasma levels of VLDL. Such results suggest that under normal conditions Tregs positively modulate VLDL cholesterol levels (7).

In summary, these studies demonstrated a link between Tregs depletion, decreased suppressive capacity and the development of atherosclerosis in mice. However, these studies failed to address key questions surrounding the Tregs roles in humans: what are the specific functions of Tregs in atherosclerosis? Why are Tregs defective in both number and suppressive function in atherosclerosis? And, most importantly, are the findings obtained in animal models applicable to humans?

## The Function of Tregs in Atherosclerosis

Subsequent studies began to address the role of Tregs within atherosclerotic pathophysiology. Tregs suppress proatherogenic Th1 effector T-cells (Figure 2). Studies in mice demonstrated the ability of Tregs to reduce the proliferation of Th1 and decrease IFNγ production via IL-10 synthesis (32). This finding has been mirrored in human CVD; compared to healthy control, patients suffering from CVD (stable angina, unstable angina, and acute MI) have a reduced CD4^+^CD25^+^FOXP3^+^ population whilst simultaneously exhibiting an increased Th1 population in circulation (33). Furthermore, another study showed the presence of increased numbers of Th17 cells and a lower proportion of Tregs present in patients with unstable carotid artery legions (34), suggesting Tregs could suppress proatherogenic Th17 effector T-cells. The existence of these correlations suggests the existence of a shifted balance between anti-inflammatory Tregs and pro-inflammatory Th17/Th1 in patients with atherosclerosis, favoring the latter subsets.

Tregs have also been demonstrated to act directly on monocytes inhibiting their cytokine secretion, differentiation, and antigen-presenting function. Following co-culture with Tregs, monocytes exhibit classical features of M2 macrophages such as increased CD206 (mannose scavenger receptor) and CD163 (hemoglobin scavenger receptor) expression. Simultaneously, macrophages co-incubated *in vitro* with Tregs exhibited a reduced capacity to respond to pro-inflammatory LPS as demonstrated by both decreased production of IL-6 and TNF-α and decreased NF-kB activation (Figure 2) (35). Furthermore, our group has recently reported that *ex vivo* expanded Tregs are very efficient at skewing monocytes toward a M2 tolerogenic phenotype. Of note, monocytes co-cultured with expanded Tregs showed a reduced capacity to increase detrimental IL-17 producing T-cells as compared to freshly isolated Tregs (36). This mechanism resulted from the decreased CD86 expression by Treg-conditioned monocytes. In addition to suppressing effector T-cells and favoring M2 macrophage development Tregs have been previously shown to decrease foam cell formation via downregulating both SRA and CD36 (37).

Tregs also exert effects on APCs by inhibiting antigen presentation. Tregs can inhibit APC function by the expression of cell surface molecules such as CTLA-4 and PD-L1/2. CTLA-4 expressed by Tregs, binds to and trans-internalize CD80/CD86 from APCs, diminishing the ability of APCs to co-stimulate T-cells (38). Increased mRNA levels of CTLA-4 have been associated with increased Tregs and decreased atherosclerosis (39, 40). Signaling via the co-inhibitory PD-1 (on Tregs) and PD-L1/2 (on APCs) also inhibits their activation. Mice globally deficient in either PD-1 or PD-L1/2 show aggravated atherosclerosis mediated by increased effector T-cell responses (41).

In addition to their effects on other leukocytes, Meng et al. (42) demonstrated ApoE^−/−^ mice adoptively transferred with Tregs had increased plaque stability, reducing the risk of plaque rupture by inducing collagen synthesis by M2 macrophages (42). Tregs have also been shown to suppress EC activation and cholesterol metabolism. pTregs suppress both TNFα and IL-1β mediated E and P-selectin expression by ECs (43).

## Tr1 and Th3 in Atherosclerosis

The role of Tr1 or Th3 cells in atherosclerosis and therefore their therapeutic potential is currently unclear. To date two studies using distinct mice models have evaluated the role of Tr1 in the pathogenesis of atherosclerosis.

Although no improvement in disease progression was observed following adoptive transfer of the *in vitro* expanded Tr1, ApoE-deficient mice immunized with OVA/CFA or mice treated with intranasal HSP60 (44) showed a marked reduction in atherosclerotic plaques size following Tr1 cell infusion (45). Furthermore, a recent study showed the frequency of Tr1 cells, IL-10 and LAG-3 expression by Tr1 cells was lower in patients with coronary artery disease as compared to healthy controls (46). However, there were no observed differences in suppression of Teffs proliferation after incubation with Tregs from patients or healthy subjects (46). Therefore, new studies are urged to better elucidate the role of these subsets of Tregs in CVD and atherosclerosis.

## Immunometabolism of Tregs in the Context of Atherosclerosis

Immunometabolism is an emerging field that investigates the interplay between immunological and metabolic processes. In addition to their exogenous antigen providing role, the contributions of microorganisms to atherogenesis are now beginning to be elucidated. Several factors including environment, diet, medication, genetics, and pathology affect the dynamic composition of the microbiota. Microorganisms produce various metabolites and nutrients such as vitamins and short-chain fatty acids (SCFA) which in turn can influence Treg generation, function, and trafficking (47). Such metabolites can be either systematically disseminated within the bloodstream for example endotoxin LPS; high levels of which is associated with cardiometabolic disorders and inflammation (48), conversely, they can remain in-suit at the site of production acting locally (48).

Butyrate is a SCFA produced by the fermentation of dietary fiber and is highly enriched in the colon (49). SCFAs are known to promote Tregs differentiation via several mechanisms; of particular note via their action as histone deacetylase (HDAC) inhibitors they are able to maintain the acetylation of the FOXP3 promoter at CNS1 and CNS3 which confers increased expression (47). Specifically, in the setting of atherosclerosis, it was demonstrated that gut-colonization of germ-free ApoE mice with strains of bacteria which differed in butyrate production; could affect the progression of atherosclerosis. Data indicated the presence of bacterial genus *Roseburia*, which is associated with high butyrate production, inversely correlated with atherosclerotic lesion size (49). However, this study failed to find significant differences between Treg populations in either the spleen or para-aortic lymph nodes from mice colonized with the two different communities (49). Further investigation will be required to fully understand the contribution of such factors to human atherosclerosis.

It is not only SCFA which have been associated with atherosclerosis; evidence suggests that certain vitamin intake is beneficial in preventing CVD. Vitamin A, C, E, and K deficiencies are all associated with increased CVD (50). Dietary sources of vitamin A are mainly retinol and retinyl esters from animal origin or, from plant sources provitamin A carotenoids which comprise numerous isomers of β-carotene (51). Vitamin A is metabolized into all-trans retinonic acid (RA); which is important for Treg development in the gut (52). Results in ApoE^−/−^ mice show that a vitamin A deficient diet resulted in an increase in both plasma cholesterol concentration and atherosclerotic lesion size as compared to healthy controls (51). Furthermore, dietary fortification with β-carotene protected against both elevated plasma cholesterol and increased lesion size in mice fed a vitamin A-deficient diet (51). The mechanisms by which β-carotene protected against these adverse outcomes was unclear. However, results in atherosclerotic patients who received 25,000 IU retinyl palmitate per day for 4 months showed an increased expression of FOXP3 in phytohemagglutinin-activated cells as compared to both healthy controls and patients receiving placebo (53), supporting the existence of a link between dietary habits, Tregs, and atherosclerosis.

## The Effect of Statins on Human Tregs

Tregs act to prevent atherosclerosis in a range of manners. Although currently no Treg therapies for atherosclerosis exist, some existing treatments have beneficial effects on Tregs. Statins are one of the most widely prescribed treatments for atherosclerosis due to their capacity to reduce cholesterol biosynthesis, it has been reported that these drugs can have other athero-protective effects.

Both atorvastatin and paravastatin attenuate T-cell activation, proliferation, inhibit the secretion of the pro-inflammatory cytokines and enhance secretion of anti-inflammatory cytokines (54). These statins inhibit IFN-γ production, which reduces Th1 activation (54), they also bind to lymphocyte function associated antigen-I (LFA-1) preventing leukocyte adhesion to ECs (54). Both mechanisms could be attributed to the indirect effect statins on Tregs (54). Indeed, both paravastatin and atorvastatin increases Treg numbers, which contributes to down-modulation of IFN-γ producing Th1 cells and reduction on EC activation. Atorvostatin treatment of human cells resulted in increased numbers of CD4^+^CD25^+^FOXP3^+^ Tregs *in vitro*, in addition to enhancing FOXP3^+^ expression (54). Similar results were reported with the used of pravastatin, which increased the number of CD4^+^CD25^+^ cells in humans. Moreover, simvastatin potentiates *ex vivo* Treg expansion.

Therefore, the benefits of statins can be partially attributed to their effects on Tregs. However, no direct analyses of the statins on Treg function and gene expression has been made, nor have how statins affect sub-populations of Tregs in humans been investigated.

## The Bidirectional Relationship Between Tregs and Risk Factors

Further to the Treg beneficial effects in atherosclerosis, disease risk factors, such as hyperlipidemia and hypertension, also affect Treg numbers and functions. Indeed, hypercholesterolemia changes plasma membrane dynamics of leukocytes, which supports the proliferation of activated T-cells as well as the size and function of the Treg cell population (55). ApoE^−/−^ mice have reduced numbers of thymic Tregs and express lower levels of CD25 concomitant with an increase in effector T-cell numbers. Moreover, Tregs from these hyperlipidemic mice are less effective at suppressing Teffs *in vitro* as compared to their WT counterparts (56).

The link between hypertension and the adaptive immune system has long been established. There is strong evidence in literature indicating that innervation of the lymphoid organs provide a pathway for direct modulation of blood pressure (57). Additionally, athymic and Rag1 deficient mice do not have increases in blood pressure after treatment with Angiotensin II or deoxycorticosterone acetate (DOCA) as compared to controls (58). The role of Tregs specifically was shown in a hypertensive rat model; Treg depletion resulted in higher blood pressure values and aggravation of cardiac hypertrophy (57). Furthermore, adoptive transfer of FOXP3^+^ Tregs protected against AngII induced hypertension (57). Accordingly, a recent study developed in our laboratory confirmed such finding and revealed Nox2 deficient Tregs are more potent in inhibiting blood pressure increases and heart fibrosis as compared to WT Tregs (59).

Extensive studies evaluating human Tregs in the context of hypertension are yet to be undertaken. Only one study published in 2018 exists, in which authors reported the down-regulation of Helios^+^ Tregs in hypertensive patients as compared to their normotensive counterparts (60). Additionally, CD4^+^ T-cells from hypertensive patients have lower FOXP3 mRNA levels than cells from healthy controls (60). This data might suggest that the hypertensive microenvironment can negatively impact Tregs populations.

Studies using Tregs in setting of both hypertension and hyperlipidemia indicate that, despite Tregs being athero-protective, they are decreased in frequency and functionality in patients suffering from these conditions, which favors the progression to CVD.

## Is Treg Therapy a Possibility for Atherosclerosis?

As a result of extensive evidence indicating the beneficial role of Tregs in atherosclerosis, there is an increasing interest regarding the potential use of these cells for immunomodulation. The use of exogenously expanded and functional Tregs may prove useful in combatting both the defective cell functionality and decreased frequency observed in atherosclerosis. Such therapy has previously been shown to be effective with no significant adverse effect in other diseases including transplantation (61), graph vs. host (61), and autoimmunity in the setting of diabetes melitus (62). The lack of adverse effects is largely attributed to the use of the recipient own cells preventing the elicitation of a detrimental auto-immune response or organ-rejection (61).

Despite such positive observations, prior to the use of exogenously expanded Tregs for treatment of human CVD and atherosclerosis further investigation is needed. A recent study demonstrated that Tregs from mice which had undergone a non-reperfused myocardial infarction exhibited defective capacity to suppress *in vitro* Teff proliferation (63). Such finding highlight that the expansion of the global autologous Treg population may not be an appropriate method to use to halt progression of such diseases. Instead a more tailored approach may be needed; in this regard, our group have published some studies those suggest the use of engineered Tregs, those exhibits a higher suppressive ability (59, 64, 65).

## Clinical Trials

Despite increasing interest in the role of Tregs in atherosclerosis, only a few clinical trials have begun to investigate their significance.

In 2010, a randomized interventional clinical trial by Del Core at Creighton University (NCT01183962) was initiated with the goal of evaluating the potentially beneficial role of oral vitamin D supplementation in patients aged 30–80 with a history of CVD in order to prevent detrimental cardiovascular events. Patients were divided into two groups, one receiving a daily oral dose of 3,000 IU of vitamin D, the other receiving no treatment. The primary endpoint was the analysis of Treg suppressive function, which was expected to improve, independently of cell number. Unfortunately, this study was terminated due to slow enrollment and funding difficulties.

In 2016, Prof. Didier Ducloux at Center Hospitalier de Besançon started the ORLY-Est trial (NCT02843867), an observational prospective study based on the immuno-monitoring of renal transplanted patients for atherosclerotic complications occurring 5 or 10 years' post-operatively. The hypothesis was that by evaluating the percentage of Tregs a prediction could be made about the likelihood of atherosclerotic complications occurring. A value under the median would be associated with a higher incidence of atherosclerotic complications by 5%. This observational study is expected to lead to a second trial (ORLY-IS) to test the effect of Treg expansion on the incidence of detrimental atherosclerotic events after transplantation.

In 2017, the group led by Johann Motsch at University Hospital Heidelberg, designed the LeukoCAPE-2 trial (NCT03105427), an observational case-only study to evaluate the use of Tregs to predict the cardiovascular risk in patients with known CVD undergoing major non-cardiological surgery, and those post cardiovascular surgery. Overall, 233 patients were enrolled, and blood was drawn at pre-defined time points up to 3 days post-operatively. Clinical follow up for cardiovascular events was carried for 30 days post-surgery. The primary outcome was the occurrence of cardiac death and/or MI and/or mL and/or myocardial injury after non-cardiac surgery (MINS) and/or embolic stroke and/or thrombotic stroke. To date, the trial is completed, but results are not yet published.

In 2019, the group led by Prof. Hongwei at Beijing Friendship Hospital started an observational, prospective trial (NCT03939338) which aims to evaluate whether the combination of both Treg frequency and cardiac magnetic resonance imaging (CMR) can be used to predict the severity of reperfusion injury following MI. The study is expected to be complete by 2021.

## Immunological Targets Within Atherosclerosis

Several studies using animal models have investigated the potential of producing preventative vaccines for atherosclerosis. Analysis of mRNA from ApoE^−/−^ mice indicates T-cells within atherosclerotic lesions show the preferential expression of a limited number of TCR-variable gene segments suggesting that a limited set of antigens are responsible for the specific T-cell response present in atherosclerosis (66). Most of the identified antigens present in atherosclerosis are generated via the modification of self-molecules; previous studies in mice have investigated the potential of some of these antigens as candidate for the production of vaccines.

OxLDL has been investigated as a candidate antigen, the generation of mucosal tolerance against oxLDL was achieved via its oral administration in LDLR^−/−^ mice prior to the onset of atherosclerosis. Oral administration attenuated both the initiation and progression of the disease. Furthermore, increased numbers of Tregs specific for oxLDL were observed in both the spleen and lymph nodes following immunization (39). ApoB100 is the peptide component of LDL and is displayed on the surface of APCs via MHCII molecules following proteolytic processing (9). Continuous sub-cutaneous infusion of ApoB100 derived peptides in ApoE^−/−^ mice resulted in reduced atherosclerotic plaque development, in addition to inhibiting the progression of previously established disease and promoting features of plaques healing such as increased collagen content, and decreased T-cell infiltration (67). Evidence indicates that the mucosal administration of the ApoB100 antigen induces antigen-specific tolerance through the generation of several Treg subsets which could be responsible for the observed athero-protective effects.

HSPs have also been found to act as antigens in atherosclerosis (40). HSP60-specific T-cells are mainly Th1 and thus have a proatherogenic phenotype and produce cytokines such as IFNγ and IL-12. Studies using LDLR^−/−^ mice have shown that induction of oral tolerance to HSP60 results in attenuated atherosclerosis which is attributed to an increased CD4^+^CD25^+^FOXP3^+^ Tregs population in both lymphoid organs and the atherosclerotic lesion. This is accompanied by an increase in HSP60-specific TGFβ and IL-10 production in the mesenteric lymph node cells (40).

These findings in mice indicate several potentially good candidate antigens for the generation of a targeted vaccine. In addition to the aforementioned antigens, there are many others associated with atherosclerosis including collagen, fibrinogen, advance glycation-end products (AGE), (LP(a)), lipoprotein-lipase (LPL), and microbial antigens (9) which have not been explored in the context of targeted therapeutics, and so their potential in the generation of either a preventative vaccine or potentially antigen-specific Tregs for uses as therapeutic treatment in atherosclerosis remains unknown.

## Discussion

Despite the increasing global burden of patients with atherosclerosis, a curative therapy is still to be found. Symptom and lifestyle management can act to slow the disease progression but ultimately it will not be totally halted due to its association with aging and vessels inflammation.

Tregs have been closely associated with atherosclerosis in both animal models and humans, with their presence and their mechanisms of action shown to be atheroprotective. Despite this evidence, there has been little investigation into the potential of Treg therapy. The few trials focusing on CVD patients and Tregs have tended to monitor Treg number, function and subtype for potential use as biomarkers for disease severity. One trial did try to utilize Vitamin D to enhance endogenous Treg populations, however this is far from mimetic a cellular therapy involving infusion of exogenously expanded autologous Tregs. As a result, many questions remain surrounding the potential use of Tregs in atherosclerosis and other chronic inflammatory diseases involving the cardiovascular system.

The production of antigen-specific Tregs is an attractive option. Indeed, such technologies are being utilized in pre-clinical models of transplantation (64, 68). However, the suppressive efficiency, stability and migratory capacity of genetically engineered Tregs need further evaluation before they can be used in the clinic.

In summary, Tregs present very promising targets with a great deal of potential. However, as a new and emerging field, it is important to carefully find a safe and efficient method for such a cellular therapy. Once achieved, Treg therapy could potentially become a viable treatment option in the battle against atherosclerosis and CVD.

## Author Contributions

GG participated in manuscript writing. CA and SCT contributed to manuscript writing, figure development, and manuscript editing. GL and CS contributed to manuscript editing.

### Conflict of Interest

The authors declare that the research was conducted in the absence of any commercial or financial relationships that could be construed as a potential conflict of interest.
